# Understanding activity and selectivity of metal-nitrogen-doped carbon catalysts for electrochemical reduction of CO_2_

**DOI:** 10.1038/s41467-017-01035-z

**Published:** 2017-10-16

**Authors:** Wen Ju, Alexander Bagger, Guang-Ping Hao, Ana Sofia Varela, Ilya Sinev, Volodymyr Bon, Beatriz Roldan Cuenya, Stefan Kaskel, Jan Rossmeisl, Peter Strasser

**Affiliations:** 1 Department of Chemistry, Chemical Engineering Division, Technical University Berlin, Berlin, 10623 Germany; 2 Department of Chemistry, University of Copenhagen, Universitetsparken 5, Copenhagen, 2100 Denmark; 3 Department of Inorganic Chemistry, Technical University Dresden, Dresden, 01062 Germany; 4 Institute of Chemistry, National Autonomous University of Mexico, Mexico City, 04510 Mexico; 5 Department of Physics, Ruhr University Bochum, Bochum, 44801 Germany; 6 Interface Science Department, Fritz-Haber-Institut der Max-Planck Gesellschaft, 14195 Berlin, Germany

## Abstract

Direct electrochemical reduction of CO_2_ to fuels and chemicals using renewable electricity has attracted significant attention partly due to the fundamental challenges related to reactivity and selectivity, and partly due to its importance for industrial CO_2_-consuming gas diffusion cathodes. Here, we present advances in the understanding of trends in the CO_2_ to CO electrocatalysis of metal- and nitrogen-doped porous carbons containing catalytically active M–N_*x*_ moieties (M = Mn, Fe, Co, Ni, Cu). We investigate their intrinsic catalytic reactivity, CO turnover frequencies, CO faradaic efficiencies and demonstrate that Fe–N–C and especially Ni–N–C catalysts rival Au- and Ag-based catalysts. We model the catalytically active M–N_*x*_ moieties using density functional theory and correlate the theoretical binding energies with the experiments to give reactivity-selectivity descriptors. This gives an atomic-scale mechanistic understanding of potential-dependent CO and hydrocarbon selectivity from the M–N_*x*_ moieties and it provides predictive guidelines for the rational design of selective carbon-based CO_2_ reduction catalysts.

## Abstract (web summary)

Inexpensive and selective electrocatalysts for CO_2_ reduction hold promise for sustainable fuel production. Here, the authors report N-coordinated, non-noble metal-doped porous carbons as efficient and selective electrocatalysts for CO_2_ to CO conversion.

## Introduction

Direct electrochemical reduction of CO_2_ (CO_2_RR) is a promising early-stage technology to produce commodity chemicals and synthetic fuels. Electricity from renewable sources can provide the input power needed to react water and waste CO_2_ to produce carbon-based chemicals or fuels in a sustainable manner^1^. The ultimate technological viability of this process, however, is contingent upon the identification of affordable catalyst materials that overcome the challenges regarding the poor product selectivity and poor voltage and energy efficiency^2^.

Metals have been the most common choice as electrocatalysts for the CO_2_RR. In early studies, copper was found to be the unique metal able to reduce CO_2_ into relevant amounts of hydrocarbons^3^. This is why catalysis studies—whenever hydrocarbon products were of primary interest—have invariably focused on Cu or Cu-derived materials. From these we now know that the reaction conditions such as electrolyte^4–6^ and applied potential^3, 7^ can have a significant effect on activity and selectivity of Cu during the catalytic reaction process. In addition, more recent work evidenced that the morphology of the copper electrode^8^, its oxidation state^9, 10^, the geometric shape^11–14^ and size^15^ of the Cu nanoparticles, the interparticle distance^16, 17^, as well as the presence of a second metal^18–20^ also play a crucial role for the resulting catalytic performance.

In contrast to hydrocarbon formation, the CO_2_ reduction to CO requires only two electron/proton transfers, which makes it a substantially less hindered process. The formation of CO is usually accompanied by HER resulting in syngas production, which can be used as feedstock in synthetic fuels production via the catalytic Fischer-Tropsch process. The chloralkaline electrolysis-based polyurethane and polycarbonate industries, however, strive to adopt an electrocatalytic cathodic reduction of CO_2_ to pure CO streams for production of phosgene intermediate further downstream. Such innovative CO_2_-depletion cathodes coupled to the anodic chlorine production electrode are still in early-stage research and currently require first and foremost fundamental advances in our understanding of the catalytic mechanism and the identification of suitable efficient catalysts.

It has been shown that Ag^21, 22^, Au-derived^23–28^ and bimetallic Cu–In^19^ and Cu–Sn^20^ catalysts can selectively reduce CO_2_ to CO at low overpotentials. However, despite their promising performance, alternative earth-abundant catalyst materials are desired. Molecular catalysts based on Iron-Porphyrin showed some CO_2_ to CO reactivity in DMF solution^29–31^, and so did metal-organic frameworks^32^ and immobilized porphyrins^33–35^. Unfortunately, these material concepts severely suffer from low electric conductivity and hence are not suitable as CO_2_ reduction catalysts at large current densities.

A promising recent alternative to expensive noble metals are solid doped carbon-based powder catalysts, similar to those developed for oxygen reduction reaction in recent years^36–40^. In recent studies, metal-free, nitrogen-doped carbon catalysts (N–C) have been proven capable to efficiently reduce CO_2_ to single- and multi-carbon species and both experimental and computational studies have pointed toward pyridinic-N as the active site^41–46^. More recent studies evidenced that the metal centers are in fact crucial for the CO_2_RR to CO. Varela et al.^33^ have tested the PANI-derived catalysts as CO_2_RR catalyst and shown that the addition of metal resulted in a strongly enhanced CO_2_RR activity and the generation of CO. More interestingly, trace amounts of CH_4_ were detected^33^. Consistently, DFT studies on transition metal based porphyrin-like catalysts suggested that depending on the metal center, *CO can be further reduced^47, 48^. A detailed fundamental mechanistic understanding of the CO_2_ reduction reactivity and selectivity of single-site metal-nitrogen-doped carbons is still missing. This contribution will change that.

Here, we explore an entire family of single site, N-coordinated transition metal-doped nanoporous carbon materials (henceforth referred to as M–N–C catalysts) as single-site electro-catalysts for the CO_2_RR. Using a combined experimental and computational approach we investigate the catalyst activity and product efficiency (catalyst selectivity) and unravel their mechanistic origins in terms of binding energies and energetic reaction paths. This family of M–N–C materials comprises highly accessible and homogeneously dispersed M–N_*x*_ sites, displays balanced surface wettability and low valence metal species, which exhibit impressively high activity and remarkable selectivity at low over-potential for the CO_2_RR to CO and hydrocarbons. We show that these catalysts are comparable alternatives to Au-based catalysts in future industrial CO_2_-consumption gas diffusion cathodes (CCCs). Density functional theory (DFT) calculations offer first-of-its-kind mechanistic insight into the rate- and selectivity determining processes on the single-site metal-nitrogen centers. We show that the binding energies of intermediates to the M–N_*x*_ moieties provide excellent descriptors to predict, and understand the mechanistic details of the CO_2_RR activity and selectivity of this family of catalysts over a wide overpotential range.

## Results

### Synthesis and characterization

**Fig. 1:**
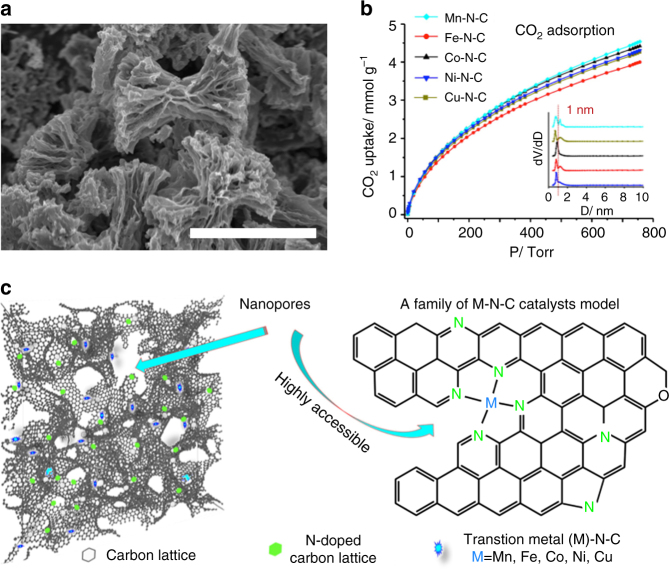
Visualization, porosity and illustration of the M–N–C catalyst. **a** Typical SEM image of the family of N-coordinated metal-doped (M–N–C) carbon electro-catalysts, scale bar=4 μm; **b** CO_2_ physisorption isotherm (273 K); inset: the pore size distribution; **c** Materials model and a schematic local structure

We have synthesized a family of M–N–C electrocatalysts starting with bipyridine-based coordinated polymers and a variety of transition metals such as Mn, Fe, Co, Ni, and Cu. Materials characterization started with morphological and gas adsorption experiments (Fig. 1a, b). The M–N–C electrocatalysts showed hierarchical chemical structures with visible macropores (Fig. 1a, Supplementary Fig. 1). The pore size distribution peaks narrowly at ca. 0.7–0.8 nm (2.5–2.9 times of the dynamic diameter of CO_2_ molecules, Fig. 1b inset), enabling this family M–N–C materials a remarkable 4.0–4.5 mmol g^−1^ capacity for CO_2_ capture at atmospheric pressure (Fig. 1b) due to their high-adsorption potential to trap CO_2_ molecules^49^. This could result in CO_2_ enrichment within a local environment despite the low CO_2_ solubility in the working electrolyte. Figure 1c displays a structural illustration of the interconnected macropore walls, composed of thin carbon branches with highly accessible micropores, all over which the coordinated metal sites as well as N-containing carbon lattice are homogeneously distributed.

The N_2_ physisorption isotherms (Supplementary Fig. 2) are essentially type I for Cu, Co, Ni, or Mn–N–C samples, indicating their microporous nature, while the visible hysteresis of Fe–N–C material reveals the presence of a small fraction of mesopores in addition to micropores. Notably, a significant increase of gas uptake was observed at higher relative pressure (*P*/*P*_0_ > 0.9) for all M–N–C samples, indicating their rich macroporosity, which is consistent with the SEM images (Fig. 1a). The specific surface area based on Brunauer–Emmett–Teller (BET) theory is in range of 615–938 m^2^ g^−1^, while the Ni–N–C and Mn–N–C show the lowest and the highest BET surface area, respectively, and the others are in between (Supplementary Table 1). This shows a roughly linear relationship with the double layer capacity under the electrochemical condition (Supplementary Fig. 3, Supplementary Table 1). The M–N–C samples showed a moderate hydrophilic character (Supplementary Fig. 4) and comparable defect site density (Supplementary Fig. 5, Supplementary Table 2). The XRD patterns (Supplementary Fig. 6) reflect the predominant amorphous carbon support, particularly for Mn, Co, Ni or Cu–N–C; while the presence of Fe, to some extent, led to graphitic domains. Some residual Fe, Co, Ni in a metallic state was detectable after the H_2_ reduction at 900 °C. The STEM elemental mappings (Supplementary Fig. 7) are fully consistent with the XRD findings showing presumably carbon-encapsulated metal particles as well as coordinated metal ion sites for the three catalysts.

### Catalyst surface

**Fig. 2:**
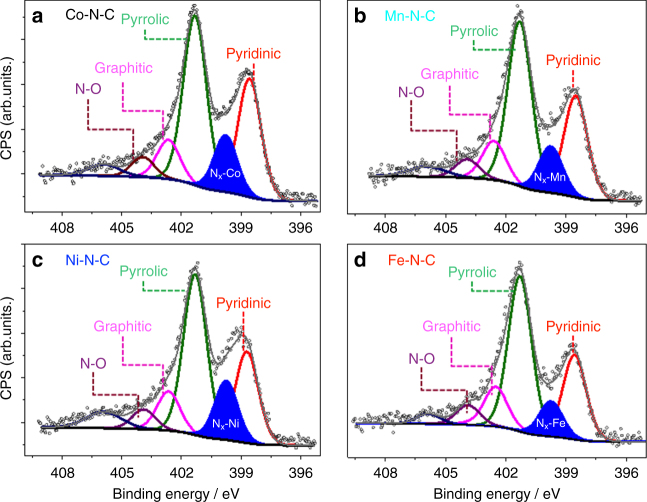
High-resolution XPS characterization. N-1s XPS core level region of **a** Co, **b** Mn, **c** Ni and **d** Fe doped M–N–C catalyst. The 2p_3/2_ spectra of the corresponding metal peaks (Co-2p, Mn-2p, Ni-2p, Fe-2p) is shown in Supplementary Fig. 9

The catalyst surface chemical composition and state were investigated using X-ray photoelectron spectroscopy (XPS). Fitted high-resolution N 1 s spectra (Fig. 2 for Co, Mn, Ni, Fe, and Supplementary Fig. 8 for Cu, detailed fitted parameters in Supplementary Table 3) evidenced the presence of the porphyrin-like metal-coordinated M–N_x_ moieties (399.7 eV), as well as pyrrolic (401.3 eV), pyridinic (398.6 eV), graphitic (402.5 eV), and N–O_*x*_ (403.9 eV) species (Fig. 2)^50, 51^. In addition, a weak and broad peak can be fitted at higher binding energies, centered at 405.9 eV, which is likely assigned to trace amounts of non-decomposed nitrogen precursors^52^. The N 1 s spectra of all samples are dominated by pyrrolic nitrogen (Supplementary Table 3), whereas the M–N_*x*_ moiety gives rise to the most intense core level for the Ni-doped sample. A detailed analysis of the metal 2p_3/2_ shake-up photoemission lines (insets of Fig. 2) offering insight in the chemical state of the metallic species is presented in Supplementary Fig. 9. Combined, our materials characterization confirmed the prevalent presence of N-coordinated metal single-site moieties, M–N_*x*_, near the surface in all catalyst samples, except for the Cu sample that exhibited evidence of near-surface metallic Cu particles (Supplementary Fig. 8).

### Catalytic sensitivity towards CO_2_

**Fig. 3:**
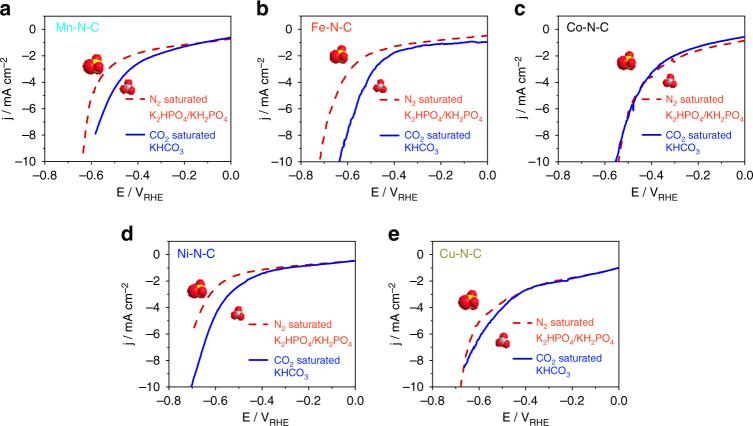
CO_2_ reduction reaction activities. Linear sweep voltammetry of **a** Mn–N–C, **b** Fe–N–C, **c** Co–N–C, **d** Ni–N–C and **e** Cu–N–C in CO_2_-saturated 0.1 M KHCO_3_ (solid lines) and in N_2_-saturated 0.1 M KH_2_PO_4_/K_2_HPO_4_ (dashed lines) with a catalyst loading of 0.76 mg cm^−2^ at 5 mV s^−1^ in cathodic direction

As a first test of the total faradaic reactivity of our single-site solid catalysts in CO_2_-saturated 0.1 M KHCO_3_, comprising both the HER and CO2RR, Linear Sweep Voltammetry (LSV) were performed between 0.0 and −0.7 V vs. RHE, blue solid curves in Fig. 3. Comparison with LSVs performed in absence of CO_2_ (red dashed curves) revealed substantial CO_2_RR activity of the Mn, Fe, Ni, and Cu-doped catalysts. Furthermore, the Mn, Fe, Ni, and Cu-doped samples exhibited an earlier onset (smaller overpotential) for the CO_2_RR than HER, suggesting that, at least in a small potential window, they are selective towards CO2RR. By contrast, Co–N–C presented a comparable activity suggesting that the HER may be the dominant faradic process over the investigated potential range.

Catalytic performance tests were conducted under potential control during 1 h electrolysis. The geometric electrode area-normalized (j_geo_) and the active interfacial area-normalized (double-layer capacity-normalized) faradaic currents (j_DL_) after 15 min and 60 min are compared in Supplementary Fig. 10. The Co–N–C catalyst generated the most overall faradaic current, while the Cu–N–C displayed the poorest overall reactivity at larger overpotentials, in accordance with Fig. 3.

### Faradaic efficiencies and absolute yields

**Fig. 4:**
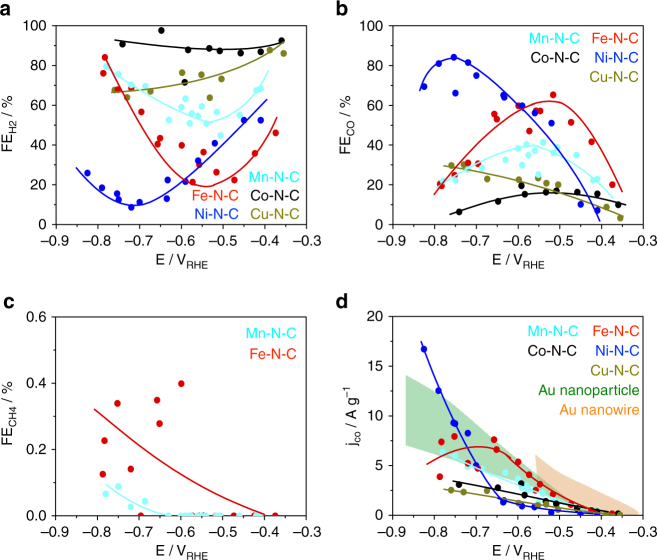
Catalytic performance and product analysis. **a**–**c** Faradaic Efficiencies (FE) vs. applied, IR-corrected electrode potential of **a** H_2_, **b** CO and **c** CH_4_. **d** Catalyst mass-normalized CO partial currents (mass activity) vs. applied potential for the five M–N–C catalysts compared to state-of-art Au catalysts (performance ranges of Au-nanoparticle and Au-nanowires are shown by filled areas^25–27^. Lines to guide the eye. Conditions: 60 min at constant electrode potential in CO_2_-saturated 0.1 M KHCO_3_ with 0.76 mg cm^−2^ M–N–C catalysts loading. Faradaic efficiencies and CO yields after 15 min are shown in Supplementary Fig. 11

The stationary faradaic efficiencies (FE) of the three principal CO_2_RR products after 60 min electrolysis are displayed in Fig. 4. H_2_ and CO accounts for up to 95% of the transferred charge on the single-site catalysts(see Supplementary Eqs. 1-3. Product distribution at 15 min is shown in Supplementary Fig. 11). Remarkably, small amounts of methane were detected, however only on Fe and Mn catalysts, while no liquid product could be detected. Despite the low number of active surface single-sites on the M–N–C catalysts, their mass-based CO partial currents (production rate) are comparable to that of Au-based catalysts^25, 26^, especially at technologically interesting higher currents (Fig. 4d). These results highlight the significance of this family of compounds as non-precious, earth-abundant low-cost and efficient CO_2_RR catalyst alternatives for the electrochemical production of CO in CO_2_-consuming electrodes.

To arrive at a fundamental mechanistic understanding of the CO_2_ catalysis on the single-site materials, we focus on reactivity trends among the M–N–C catalysts at different applied overpotentials. The CO_2_RR performance tests exhibit a strong dependence on the nature of the transition metal, not only in terms of the molar CO/H_2_ ratio, but also in the experimental overpotential at maximum CO efficiency (see Fig. 4a and b). H_2_ FE on Co–N–C remains above 80% over the entire electrode potential range, making it a catalyst with poor selectivity towards CO_2_RR. On the other hand, Fe–N–C and particularly Ni–N–C catalysts clearly act as highly promising catalyst for selective CO production, however, the maximum CO FE is obtained at a smaller overpotential on Fe–N–C (V_RHE_ = −0.55 V, FE_CO_ = 65%) than on Ni-N-C (V_RHE_ = −0.78 V, FE_CO_ = 85%). Note that the selectivity of these two single site catalysts is drastically different from that of metallic Ni and Fe catalysts, which yield H_2_ as virtually the only major product^23^. We have conducted a number of control measurements to confirm that the M–N_*x*_ site is indeed the most significant active center for CO_2_ reduction into CO. First, the Nitrogen Free M–C as well as the polymer precursor before pyrolysis (Cu-Bpy) contributes negligibly to the CO activity during the CO_2_RR process. Second, we could not exclude some catalytic activity of the Nitrogen functionalities. However, based on their finite CO_2_RR catalytic reactivity and the rough similarity of the distribution in the M–N–C catalysts, their effect could be seen as a known weak background signal for all cases (Supplementary Table 3, Supplementary Fig. 12 and 13). This finding strongly suggests that the CO_2_RR (to CO) reactivity trends purely originate from the differences in intrinsic catalytic activity of the various M–N_*x*_ moieties.

### Atomistic insight into the M–N–C activities

To bring theoretical mechanistic insight, DFT simulations pertaining to the CO_2_ reduction process on N-coordinated metal-doped M–N–C catalysts were carried out. For this purpose we took the single-site motif M–N_4_ as active site to calculate the binding energy of the different reaction intermediates (Supplementary Fig. 14). We note that there exist other M–N_*x*_ functionalities^53–55^; however, previously we computationally found the metal to be the dominating factor as compared to other M-N_*x*_ functionalities^56^. Thus, the M–N_4_ site appears to be a reasonable single active site model for our analysis here. For the model we calculate the binding energies without electrolyte, which is reasonable for the trends and conclusions drawn here. While activity can often be associated with a single descriptor, selectivity can obviously not, as it is related to competition between different possible reaction paths. The different reaction paths show different dependence on metal center and potential.

**Fig. 5:**
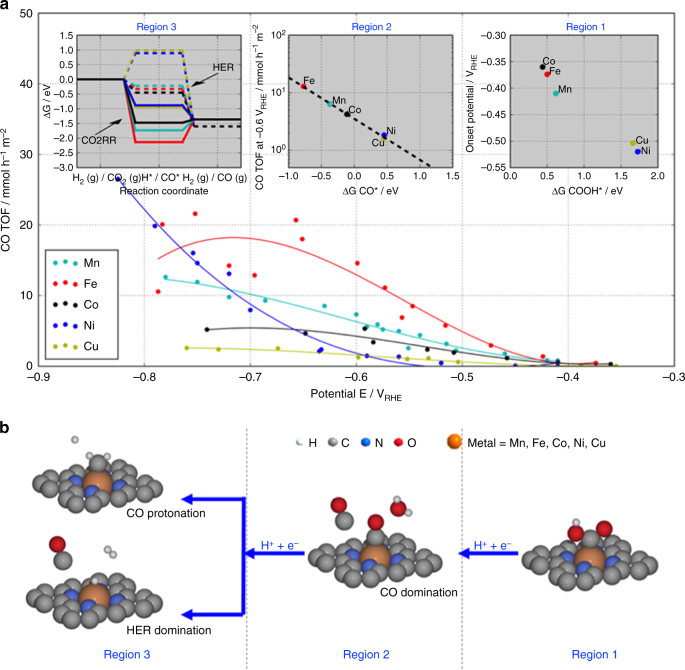
Experimental correlation to simulations. Experimental CO production turnover frequency (TOF) of the M–N–C catalysts vs. applied IR-corrected electrode potential (see Supplementary Equation 4). The catalytic reactivity trends **a** and reaction pathway **b** split into 3 potential regions with distinctly different rate-determining mechanistic features. Insets: Region 1: Low overpotentials, the experimental onset potentials of CO production (better seen on the log (CO TOF)—E plot in Supplementary Fig. 15) correlate with the binding energy of the reaction intermediate COOH* taken from Supplementary Fig. 14. Region 2: Intermediate over-potentials, CO production TOF at −0.6 V_RHE_ correlates with the free energy of adsorbed CO, CO* taken from Supplementary Fig. 14; Region 3: High overpotentials, free energy diagrams for the HER (dashed paths) and CO2RR (solid paths) at −0.8 V_RHE_ for each M–N–C catalyst. HER barriers are high for Ni and Cu, while CO2RR is downhill making these materials favorable CO producing catalysts

Figure 5 compares the trends in the experimental CO-specific turnover frequencies (TOF) of the five M–N–C catalysts. The TOF values were derived from the absolute CO production rates normalized by the respective BET surface area-weighted surface M–N_*x*_ concentration (Supplementary Equation 4). Correlating the experimental TOF trends and predicted DFT theoretical energy diagrams we were able to identify three regions of distinct reaction dynamics that control the electrocatalysis: First, Region 1, a dynamic regime at low overpotentials near the onset of the CO production, Then, Region 2, a dynamic regime at intermediate overpotentials and finally, Region 3, at larger overpotentials where the CO_2_ reduction current densities approach technologically relevant levels. The reason for this division is that the order in catalytic activity change in the different regions indicating that the rate in the different regions is determined by a distinctly different surface chemistry.

### Low overpotential regime around −0.45 V_RHE_ (Region 1)

Defining the CO production onset potential to be the applied electrode potential at which the CO TOF exceeds 0.2 mmol/(h m^2^_active_), the Fe, Mn and Co–N–C catalysts start producing CO at around −0.4 V vs. RHE, while the Cu and Ni samples require considerable higher overpotentials, see Region 1 inset in Fig. 5. The onset potential is determined by the mechanistic elementary step that is the last to become exergonic as the overpotential is increased (limiting potential). Simulations suggest that this potential-determining step is the first proton-coupled electron transfer reduction of CO_2_ to adsorbed COOH according to

1 $${\rm{C}}{{\rm{O}}_2} + {{\rm{H}}^ + } + {{\rm{e}}^ - } \to {\rm{COOH}}^*$$

In agreement with electrochemical measurements, in the simulations the catalyst falls into two groups: Co, Fe and Mn requiring only a small overpotential, whereas Cu and Ni need a larger thermodynamic driving force for that step.

### Intermediate overpotential regime around −0.6 V_RHE_ (Region 2)

Here, the Fe- and Co–N–C catalysts approach their maximum CO_2_RR reactivity, while the Ni–N–C catalyst has barely passed above its CO production onset. With the electrode potential being now past the limiting electrode potential, the overall CO_2_ reduction reaction invariably becomes limited by a non-faradaic chemical reaction step. The larger the thermodynamic driving force of this step, the faster the overall reaction rate. Correlating experiments to DFT calculations reveals that the logarithm of the experimental CO TOF is now linearly related to the CO* binding energy descriptor, see Region 2 inset in Fig. 5. This suggests that the rate-controlling intermediate has shifted from COOH* to CO*. As a result of this, the overall reaction rate appears limited by the process

2 $${\rm{COOH}}^* + {{\rm{H}}^ + } + {{\rm e}^ - } \to {\rm{CO}}^* + {{\rm{H}}_2}{\rm{O}} \cdot $$

While DFT predictions do not allow us to unambiguously pinpoint the exact rate-limiting point along the reaction coordinate of step (2), we hypothesize that it is the chemical dissociative formation of H_2_O according to COOHH*→CO* + H_2_O. The stronger CO* binds, the more driving force is available for this step. An evidence for the hypothesis can be considered by comparing the Fe- and Mn–N–C, which have almost similar COOH* and H* binding. However, these descriptors cannot explain the experimental different CO TOF from the two, while the logarithm to the CO* descriptor can. (Supplementary Fig. 15)

### Large overpotential regime <−0.7 V_RHE_ (Region 3)

Here, the experimental CO formation TOF in Fig. 5, as well as the faradaic CO efficiencies of the Fe–N–C and Co–N–C catalysts have passed their maximum and trend downward (Fig. 4), that of Mn–N–C is levelling off, while the Ni–N–C catalyst continues to increase its CO production rate at a very high faradaic CO efficiency, significantly outperforming all other single-site catalysts as well as Au catalysts.

Our mechanistic DFT analysis shown in inset (Region 3) of Fig. 5 is able to consistently explain all these experimental findings. The free energy diagrams of the HER (dashed) and CO_2_RR (solid) evidences that the Fe-, Co- and Mn-based catalysts start to strongly catalyze the hydrogen evolution reaction (H^+^ + e− → H* → H_2(g)_) illustrated by the all downhill reaction energy pathways. Among them, Co–N–C is the most efficient HER catalyst and, thus, displays the highest faradaic efficiency for hydrogen evolution, see Fig. 4. In contrast, the Ni- and Cu-based catalysts exhibit very weak binding of H* which makes the HER thermodynamically unfavorable at −0.8 V_RHE_, giving rise to low faradaic hydrogen efficiencies.

The DFT predictions of the CO_2_RR pathway (CO_2_ → CO* → CO_(g)_) at −0.8 V_RHE_ complete the mechanistic picture. While the Ni–N_*x*_ and Cu–N_*x*_ moieties stand out as the single sites with the weakest binding to COOH* and therefore with the largest overpotential to start CO_2_ reduction (see Region 1), their weak binding of CO* prevents the potential-independent chemical CO-desorption process (CO* → CO_(g)_) to become rate-limiting. This is in contrast with the Mn, Fe, Co–N_*x*_ sites whose CO TOF is controlled by the CO* → CO_(g)_ step due to their strong CO* binding (solid pathways in Region 3) leading to a positive ΔG of CO desorption. Indeed, experimentally, the CO TOF values of the Mn, Co, and Fe–N–C level off or slow down, while the hydrogen evolution accelerates.

We note that the relatively strong binding of CO* on Fe–N_*x*_ and Mn–N_*x*_ single sites predicted for Region 3 is fully supported by the experimentally confirmed exclusive ability of these two catalysts to produce the hydrocarbon CH_4_, see Fig. 4. In simple terms, one could say that to produce subsequent reaction products from CO during the CO_2_RR, the CO molecule must be bound strong and long enough to undergo subsequent dissociation and hydrogenation steps to arrive at CH_4_. For the Ni and Cu catalysts, the CO* detaching is energetically all downhill reaction which prevents further transformations. This makes Ni–N_*x*_ and Cu–N_*x*_ single-site catalysts ideal electrochemical CO producers. We note that the experimentally observed reactivity trend of the Cu–N_*x*_ catalysts in region 3 does not closely follow that of Ni. This is due to a DFT-predicted thermodynamic instability (not shown) of the Cu–N_*x*_ moiety under the strongly reducing conditions of < −0.7V_RHE_ in region 3. As a result of this, the N-coordinated Cu ions spontaneously reduce to metallic Cu nanoparticles—as confirmed by our XPS results—which show lower CO efficiency and lower TOF values at electrode potentials of region 3^3, 23^.

## Discussion

In this work, we found a family of solid, single site, N-coordinated transition metal-functionalized nanoporous carbons that show very high electrocatalytic reactivity and selectivity with respect to the direct CO_2_ reduction to CO (CO_2_RR). A technical challenge in these M—N—C catalyst is to achieve a high density of active M–N_*x*_ sites, while minimizing effects of other nitrogen moieties and inorganic metal impurities, which, for this class of materials cannot be completely excluded. However, based on our experimental observation, we could confirm that the M–N_*x*_ site play the dominant role during the CO_2_RR process into CO. For instance, the Co–N_*x*_ sites were efficient hydrogen producers whereas the Fe- and Ni–N_*x*_ single site catalysts showed a unique reactivity and faradaic efficiency for reducing CO_2_ into CO, meeting and exceeding the mass-based activity of state-of-art Au catalysts at a fraction of their cost.

To understand the trends in reactivity and selectivity of the single site catalysts we have correlated our experimental results with DFT simulations of the energetics of the competing reaction pathways involved. Our results demonstrate that the binding energies can be used as descriptors to predict the CO_2_RR activity and selectivity of this class of catalysts. This is why we find a good agreement between the DFT predictions and the catalytic experiments offering a detailed mechanistic understanding of the role of the metal centers in the considered catalytic processes.

Consistent with experiments, Co–N_*x*_ sites displayed all-downhill energetics for hydrogen, but severe energetic barriers to CO formation. By contrast, the low H* and CO* binding energy of the Ni–N_*x*_ single site required larger overpotentials to jump start the reactions. At larger overpotentials, however, Ni–N_*x*_ catalysts displayed all-downhill energetics toward CO, while hydrogen evolution is hindered.

The high CO efficiency at medium and large overpotentials of the Fe–N–C and Ni–N–C materials combined with their earth-abundant constituents, compared with standard Au catalysts, makes them attractive catalysts for deployment in future industrial CO_2_-consuming CO cathodes for use as counter electrode process in the chlorine production industry. The choice of catalyst thereby becomes a tradeoff between voltage efficiency (Fe produces most CO at lower potentials) and turnover frequency/current density (Ni makes most CO at higher overpotentials). In particular for Ni–N–C catalysts, high CO efficiencies at current densities approaching industrial levels make them suitable candidates for CO_2_-consuming gas-diffusion cathode (CCC) designs to be deployed in next-generation chloralkaline electrolyzers. By eliminating the need for fossil fuel-based steam reforming toward purified CO feed streams in the Chlorine-mediated polymer industry, CO_2_ reuse in chlorine–CO co-electrolyzers would significantly contribute to a lowering of industrial CO_2_ emissions worldwide.

## Methods

### Synthesis

All chemicals were used as received. Typically, 4,4′-Dipyridyl hydrate (bipy, 1.114 g, Sigma-Aldrich Co.) was dissolved in 100 mL ethanol solution. A certain amount of CoCl_2_·6H_2_O (1.2 g) was dissolved in 900 mL DI water solution. Then the bipy solution was mixed with a CoCl_2_·6H_2_O solution and left standing for 24 h without stirring. Then, 50 mL CuCl_2_·2H_2_O (0.1 M) solution was rapidly mixed with the bpy-Co^2+^ solution and aged for 4 h. Subsequently, the resultant product was collected by centrifugation with the speed of 4200 r.p.m. for 12 min. After drying, the polymer product was carbonized at 500 °C for 2 h at a heating rate of 60 °C h^−1^ in Ar atmosphere. Finally, hydrophilic N-doped porous carbons (N–C) with trace amounts of Cu were obtained after leaching in 4 M HNO_3_ for 24 h.

Subsequently, additional transition metal species (M=Mn, Fe, Co, Ni) were introduced in N–C through incipient impregnation of MCl_*x*_ solutions. The nominal weight concentration of M respective to N–C was set to 25 wt%. The dried M–N–C composite was re-pyrolyzed at 900 °C for 2 h at a heating rate of 2.0 °C min^−1^ in Ar atmosphere. The carbonized M–N–C was dispersed in aqueous sulfuric acid (ca. 2.0 mol L^−1^) and refluxed at 100 °C for 1 day. The leached sample was collected and washed with DI water until pH value close to neutral. Finally, the leached sample was treated at 900 °C first in Ar for 2 h, and then in H_2_ for another 1 h, then again in Ar, let cool down and harvest the final M–N–C electrocatalysis (M=Mn, Fe, Co, Ni). The Cu–N–C material was obtained directly after the reductive annealing procedure without any additional acid leaching.

### Physico-chemical characterization

Scanning electron microscope (SEM) investigations were carried out with a Hitachi SU8020 instrument. A FEI Tecnai G2 F20 microscope that was equipped with HAADF-STEM and EDAX detectors was employed to acquire the HAADF-STEM images and EDX elemental maps. XPS measurements were carried out in an ultra-high vacuum (UHV) system equipped with a monochromatic Al Kα X-ray source (1486.6 eV; anode operating at 12.25 kV and 300 W) and a high-resolution Phoibos 150 MCD analyzer (SPECS). X-ray photoemission spectra were measured in fixed analyzer transmission mode with a pass energy of 15 eV and step size of 0.5 and 0.05 eV for survey and high-resolution region scans correspondingly. The binding energy scale was adjusted assigning the signal of graphitic carbon to 285 eV. Raman spectra were measured on a Renishaw Ramascope RM 2000 Raman microscope (×50, na = 0.75) with a REM Laser from Laserquantum (wavelength: 532 nm). The peak parameters were extracted by curve fitting on the Renishaw Wire 2.0 software, where a mixed Gaussian and Lorentzian function was used, and the maximum of the first curve is defined on 1170 cm^−1^. Nitrogen sorption isotherms were measured on a BELSORP adsorption analyzer at liquid nitrogen temperature. The specific surface area was calculated based on adsorption points in the relative pressure of 0.05 < P/P0 < 0.20 according to Brunauer–Emmett–Teller (BET) theory. Pore size distributions (PSDs) were derived from the adsorption branches of the isotherms based on Non Localized Density Functional Theory (NLDFT, nitrogen on carbon slit adsorption branch kernel). Water physisorption measurements were carried out on a Quantachrome Hydrosorb 1000. CO_2_ adsorption isotherms were measured on a Autosorb iQ MP micropore analyzers at 273 K. Before gas or vapor physisorption measurements, the samples were degassed at 150 °C for at least 12 h.

### Electrochemical measurements

The electrochemical measurements were controlled with EC-Lab SP-200 Potentiostat. Resistance between reference electrode and working electrode was measured with Potential Electrochemical Impedance Spectroscopy (PEIS). 50% of the resistance was corrected by the software and the rest was manually corrected. CO_2_ electrolysis was carried out in a custom-made two-compartment cell, in which the working electrode was separated from the counter electrode by Nafion membrane to hinder the re-oxidation of the products on the counter electrode. The glassware was cleaned in nochromix mixed sulfuric acid bath and afterwards in concentrated HNO_3_ for 1 h, respectively, rinsed and sonicated with 80 °C ultrapure water several times, and dried at 60 °C in an oven. Each compartment of the cell was filled with 40 mL CO_2_ (Air liquid 4.5, flow from button, rate: 30 mL min^−1^) purged electrolyte. A Pt mesh 100 (Sigma-Aldrich 99.9%) was used as counter electrode (CE) and a leak-free Ag/AgCl electrode (Hugo Sachs Elektronik Harvard apparatus GmbH) was used as the reference electrode. The CO_2_ free measurements were carried out in N_2_-saturated 0.1 M KH_2_PO_4_/K_2_HPO_4_ (Sigma-Aldrich, pH of 6.9), while the CO_2_ electrolysis in presence of CO_2_ was done in 0.1 M KHCO_3_ (Sigma-Aldrich, pH of 6.8).

### Catalytic product analysis

After 15 min and 60 min of bulk CO_2_ electrolysis at constant working potential, a sample of the gas was analyzed by gas chromatography (Shimadzu GC 2014, Haye.Sep Q (Col-No. CS 1015-03) + Haye.Sep R (Col-No. CS 1015-07), TCD and FID detector) to quantify the instant Production Rate and Faradaic Selectivity of the gaseous products. In addition, 2 ml of the electrolyte after reaction was analyzed by high performance liquid chromatograph (HPLC Agilent 1200, Zimmer Chromatography® Column, RID detector) to measure formic acid concentration and analyzed by liquid injection gas chromatography (Shimadzu GC 2010 plus, Fused-Silica-Capillary Column, REF 72306030, FID Detector) for alcohol products.

### DFT calculations and predictions

The porphyrin-like structure was created in ASE^57^ by a 3 × 5 unit cell of graphene and functionalized by removing carbon atoms to create the metal-nitrogen site. Further, the outmost carbon atoms were fixed in position and periodic boundaries were applied. This setup is hereby almost similar to Tripcovic^47^ and revealed similar results when applying the same references. For the electronic calculations the projector augmented wave method together with spin polarization and the revised Perdew–Burke–Ernzerhof (RPBE)^58^ functional was performed with the GPAW software^59, 60^. We applied a 0.18 grid spacing together with a (2 × 2 × 1) k-point sampling and all the structure were relaxed to a force below 0.1 eV/Å. To calculate the free energy diagrams, the hydrogen electrode was employed^61^. For this we use zero point, entropy and heat capacity values from reference^62^. Finally, the CO_2_ calculated RPBE energy was corrected by 0.45 eV together with a –COH water correction of 0.25 eV and a *CO water correction of 0.1 eV^63^.

### Data availability

DFT structures and binding energies will be available online in our group webpage (Rossmeisl) upon publication. Experimental data is available from the authors upon reasonable request.

## Electronic supplementary material

Supplementary Information
