# Supplementary material for: Understanding activity and selectivity of metal-nitrogen-doped carbon catalysts for electrochemical reduction of CO2
Source: Nat Commun. 2017 Oct 16;8:944. doi: 10.1038/s41467-017-01035-z (PMC5643516; doi:10.1038/s41467-017-01035-z)
Supplement: Supplementary file 1 — Supplementary Information [file 41467_2017_1035_MOESM1_ESM.pdf]

Supplementary Tables

Supplementary Table 1. Physical parameters of nitrogen-metal doped carbon (M-N-C) CO2RR electrocatalysts.

ID	S_{BET} [a] $m^2\ g^{-1}$	DL-Cap [b] $mF\ cm^{-2}$	Overall metal by ICP wt. %		ID/IG [e]
			Metal [c]	Cu [d]	
Mn-N-C	938	87.82	Mn: 0.44	0.16	1.70
Fe-N-C	724	66.94	Ni: 2.88	0.12	1.75
Co-N-C	758	80.39	Co: 1.11	0.17	1.79
Ni-N-C	615	38.82	Fe: 1.73	0.14	1.83
Cu-N-C	807	74.38	-	1.11	1.80

a) S_{BET}: N_2 ad/desorption-based surface area; b) Double layer capacity values (Geometric area of each electrode is 1 cm^2 with 0.76 mg cm^{-2} catalyst loading); c,d) overall metal content determined from ICP-OES measurement. Notably, the detection limit of ICP-OES is as low as 0.1, 0.25, 0.4, 0.5 and 0.1 μg/L for Fe, Co, Cu, Ni and Mn, respectively, where the measured solution of the dissolved M-N-C sample is 5000, 1400, 900, 1700 and 1300 times higher than the limit, indicating a low uncertainty. Each sample is measured twice. The value in the table is the average. e) ID/IG peak ratio: Ratio of D band to G band based on the Raman spectra through peak fitting with mixed-type Lorentzian/Gaussian fit functions. See Supplementary Figure 4 for the experimental Raman spectra as well as Supplementary Table 2 for the fitting detail.

Supplementary Table 2. Fitting details of Raman spectra for this family of M-N-C electrocatalysis.

ID	Curve name	Center	Width	Height	% Gaussian	Type	Area	Peak height	Area	All area I(D)/I(G)
Cu-N-C	D2	1170	238.749	548.914	100.00	Mixed	1.40+E5	I(D1)/I(G1): 2.13 I(D2)/I(G2): 0.19 I(G2)/I(G1): 1.40	I(D1)/I(G1): 6.90 I(D2)/I(G2): 0.24 I(G2)/I(G1): 3.27	1.80
	D1	1353.87	219.245	4302.14	47.9629	Mixed	1.25+E5			
	G2	1548.15	166.307	2828.96	61.3572	Mixed	5.93+E5			
	G1	1598.05	77.7354	2015.82	81.5916	Mixed	1.81+E5			
Co-N-C	D2	1170	196.548	569.691	100.00	Mixed	1.19E+05	I(D1)/I(G1): 1.48 I(D2)/I(G2): 0.22 I(G2)/I(G1): 0.81	I(D1)/I(G1): 4.05 I(D2)/I(G2): 0.25 I(G2)/I(G1): 1.47	1.79
	D1	1348.52	226.881	4939.44	53.3781	Mixed	1.46E+06			
	G2	1536.86	157.42	2767.23	68.2247	Mixed	5.34E+05			
	G1	1598.81	90.6636	2743.43	68.4198	Mixed	3.05E+05			
Ni-N-C	D2	1170	158.217	189.839	99.4851	Mixed	3.21E+04	I(D1)/I(G1): 1.48 I(D2)/I(G2): 0.24 I(G2)/I(G1): 0.81	I(D1)/I(G1): 3.73 I(D2)/I(G2): 0.26 I(G2)/I(G1): 1.33	1.75
	D1	1350.86	198.226	1434.15	71.0305	Mixed	3.44E+05			
	G2	1540.75	147.152	783.559	100.00	Mixed	1.23E+05			
	G1	1597.85	76.094	970.745	63.685	Mixed	9.22E+04			

Fe-N-C	D2	1170	153.925	163.78	100.00	Mixed	2.68E+04	I(D1)/I(G1): 1.58 I(D2)/I(G2): 0.18 I(G2)/I(G1): 0.89	I(D1)/I(G1): 5.20 I(D2)/I(G2): 0.17 I(G2)/I(G1): 2.03	1.83
	D1	1348.91	188.192	1631.51	53.16	Mixed	4.00E+05			
	G2	1549.09	153.762	923.131	92.93	Mixed	1.56E+05			
	G1	1598.56	66.255	1034.39	88.66	Mixed	7.69E+04			
Mn-N-C	D2	1170	203.708	578.058	64.01	Mixed	1.47E+05	I(D1)/I(G1): 1.57 I(D2)/I(G2): 0.19 I(G2)/I(G1): 0.94	I(D1)/I(G1): 5.40 I(D2)/I(G2): 0.24 I(G2)/I(G1): 2.52	1.70
	D1	1348.46	193.130	5042.11	41.72	Mixed	1.32E+06			
	G2	1549.16	163.501	3009.19	62.10	Mixed	6.18E+05			
	G1	1597.1	64.802	3210.22	77.57	Mixed	2.45E+05			

Supplementary Table 3. Metal atom, nitrogen content and assignment of the different N species in the M-N-C CO2RR electrocatalysts extracted from XPS data. Total metal and nitrogen content are calculated from measured XP spectra areas using instrument-specific relative sensitivity factors provided by manufacturer (SPECS).

**Sample**	**Metal[a] atom %**	**Nitrogen[a] atom%**	**Fitting of Nitrogen moieties / atom%**					
			**M-N**	Pyridinic	Pyrrolic	Graphitic	N-O	CH-Ox
Mn-N-C	**Mn:** 0.2	8.2	**12.2**	25.8	43.9	10.3	5.0	2.8
Fe-N-C	**Fe:** 0.3	8.2	**10.4**	24.2	44.6	11.7	6.0	3.1
Co-N-C	**Co:** 0.4	10.5	**13.4**	28.1	41.6	9.8	4.4	2.7
Ni-N-C	**Ni:** 0.7	9.5	**20.2**	17.1	43.6	10.7	5.3	3.2
Cu-N-C	**Cu:** 0.8	14.6	**15.5**	30.4	32.9	7.0	3.9	10.3

Supplementary Note

Detailed analysis of the metal core-level spectra. Better understanding of the type of metallic species present in these samples can be obtained by a detailed analysis of the corresponding $2p_{3/2}$ photoemission lines, shown as insets in **Figure 2**. Due to the low metal loading in our samples, we cannot fit the data with the multiplet splitting features typical for oxides and hydroxides.[1] Instead, in order to gain insight into whether oxidized metal species or nitrogen-ligated metals prevail in our samples we have compared the main $2p_{3/2}$ photoemission peak positions with data reported for divalent metal species and metalloporphyrins, and a ratio of its area to the area of the shake-up satellites, which serves as a fingerprint of 3d metals in 2+ state. Muralidharan and Hayes have reported shake-up structures for Co(II), Ni(II) and Cu(II) porphyrins to be significantly weaker than the corresponding satellites of simple oxides of the same elements.[2] Thus, Ni(II) porphyrin exhibits an XP spectrum with $2p_{3/2}$ peak centered at 854.8 eV, which is similar to $Ni(OH)_2$, but unlike the latter it has a shake-up structure of only 11% intensity as compared to the main line. In fact, the Ni-N-C sample in our study has the main Ni $2p_{3/2}$ photoemission peak at 854.9 eV and a shake-up peak just 18% of its area, suggesting a dominant presence of Ni bound to nitrogen in our samples. In contrast, the Co-N-C sample displayed a significantly more intensive shake-up structure, ca. 20% of the main Co 2p3/2 peak area, which is considerably higher than a value reported for Co(II) porphyrin (6.5%), but also clearly lower than CoO and $Co(OH)_2$ (38% and 54% correspondingly). Mn $2p_{3/2}$ of Mn-N-C has its maximum at 641.7 eV, similar to MnO, and lower than compounds with Mn^{3+} and higher oxidation states.[3] At the same time, the shake-up structure is as high as 11% of the main photoemission peak, which is slightly higher than the one reported for MnO (9%). Unfortunately, there is no reliable XPS reference for the manganese porphyrin structure. One can speculate however that Mn-N-C clearly contains divalent Mn species with a ligand structure different from common Mn^{2+} compounds. The Cu 2p XP spectrum (Supplementary Figure 8a) shows a clear presence of divalent copper, as evident from the well-developed shake-up. Cu $2p_{3/2}$ XP spectrum shows two sharp peaks at 932.7 and 934.8 eV and a shake-up satellite structure between 938.4 and 947.3 eV. A binding energy of 934.8 eV is similar to the value reported by Biesinger at al[1] for $Cu(OH)_2$, but also to Cu porphyrin.[2,3] The observed intensity of the shake-up satellite structure is 59% of the main line, which is only slightly higher than the 53% for both, Cu-porphine[2] and $Cu(OH)_2$ reported by Biesinger[1]. The peak at 932.7eV can be assigned either to metallic copper or to Cu_2O, however a detailed analysis is not possible due to weak CuLMM Auger line.[1] It appears however reasonable to assign this peak to the Cu^+ state, since metallic copper in the near-surface region is unlikely to remain after multiple acid treatments. The Fe $2p_{3/2}$ spectrum of the Fe-N-C sample shows a broad peak at 710.8 eV with a shoulder at ca. 708 eV. The position of the former and absence of distinct satellite structure points out the presence of significant amount of Fe_2O_3, while the latter is in an agreement with the values reported earlier for iron phthalocyanine.[4] According to Fe $2p_{3/2}$ XP spectrum deconvolution, the nitrogen-coordinated iron comprises only 1.6% of the total Fe content observed by XPS.

Supplementary Figures

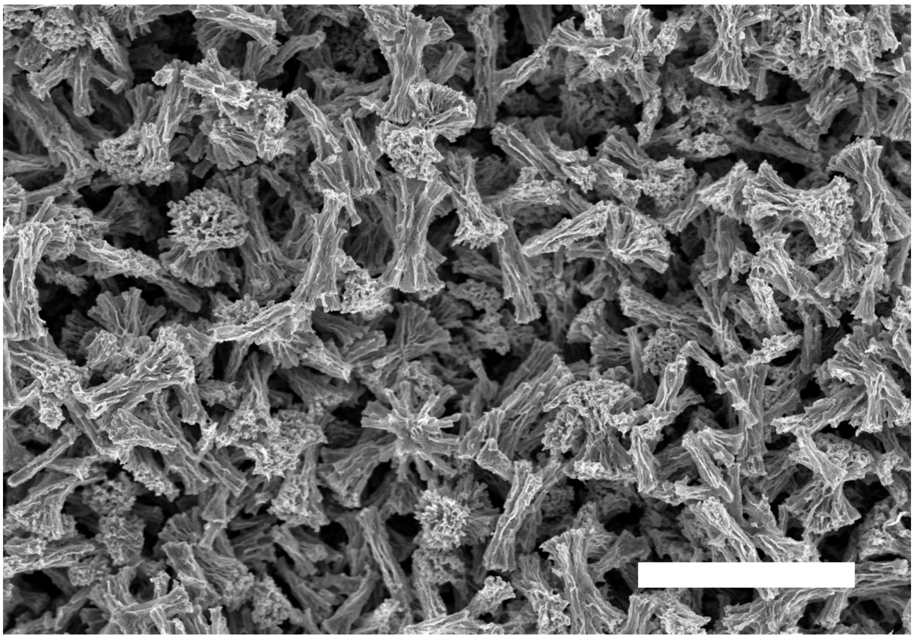

Supplementary Figure 1. Typical Scanning Electron Microscopy image of the M-N-C electrocatalysts. Scale bar: 10 μm.

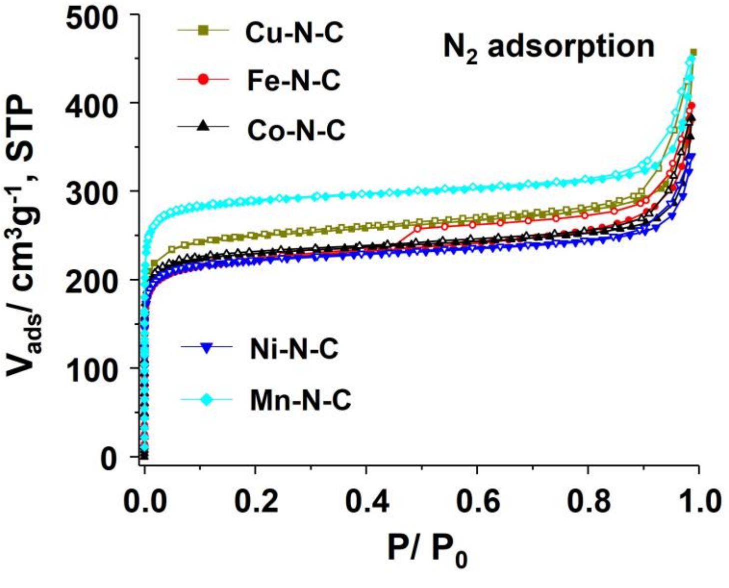

Supplementary Figure 2. N_2 physisorption isotherms for this family of M-N-C electrocatalysts. As shown, the isotherms are type I indicating the microporous structures; the abrupt increase in the higher pressure regime indicating the existence of macropores. In short, these information confirms the rich of microporosity, and indicates the hierarchical structure.

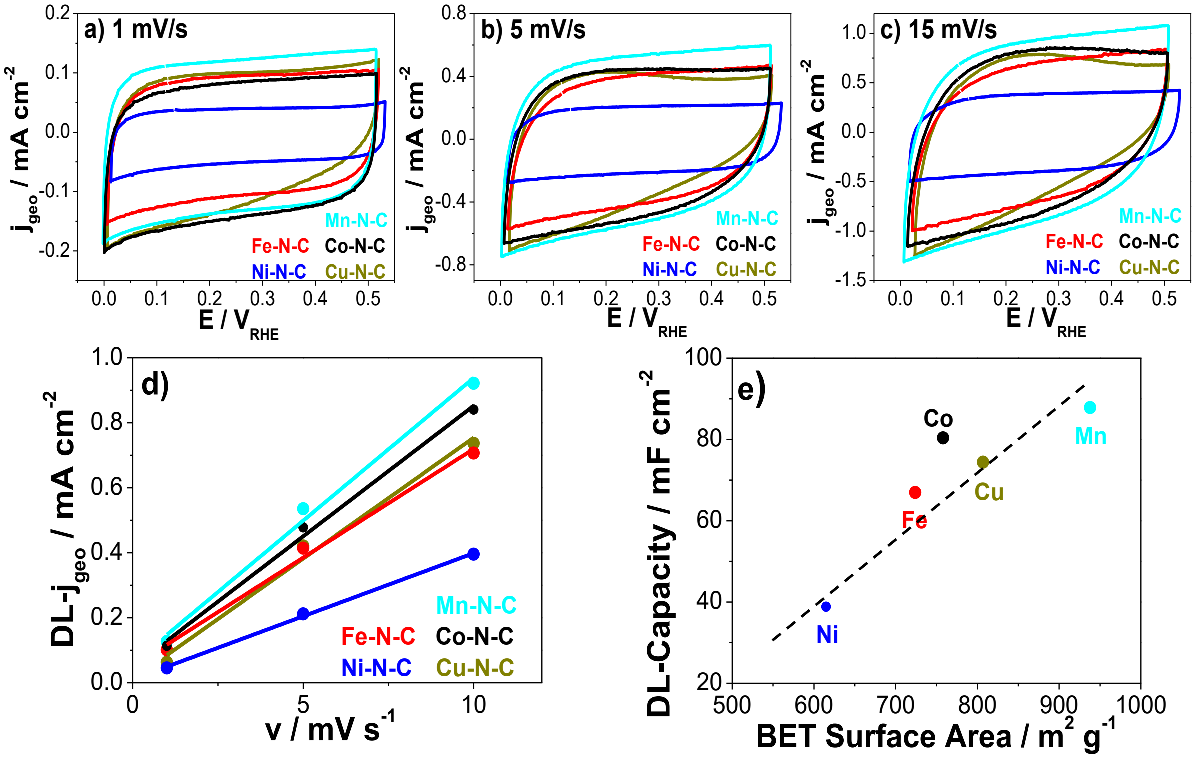

Supplementary Figure 3. a) - c) Cyclic voltammetry of the five M-N-C catalysts conducted in CO_2-saturated 0.1 M $KHCO_3$ at various scan rates for estimation of double layer (DL) capacity; d) current densities from a-c plotted vs. electrode potential scan rate to extract the double layer capacity; e) Correlation of double layer capacity and the N_2 adsorption derived BET surface area. Cycle voltammetry was performed between 0.0 and 0.52 V vs. RHE to avoid the interference of the faradaic process. Double layer current densities are utilized to determine the double layer capacity, which is proportional to the double-layer interfacial area.

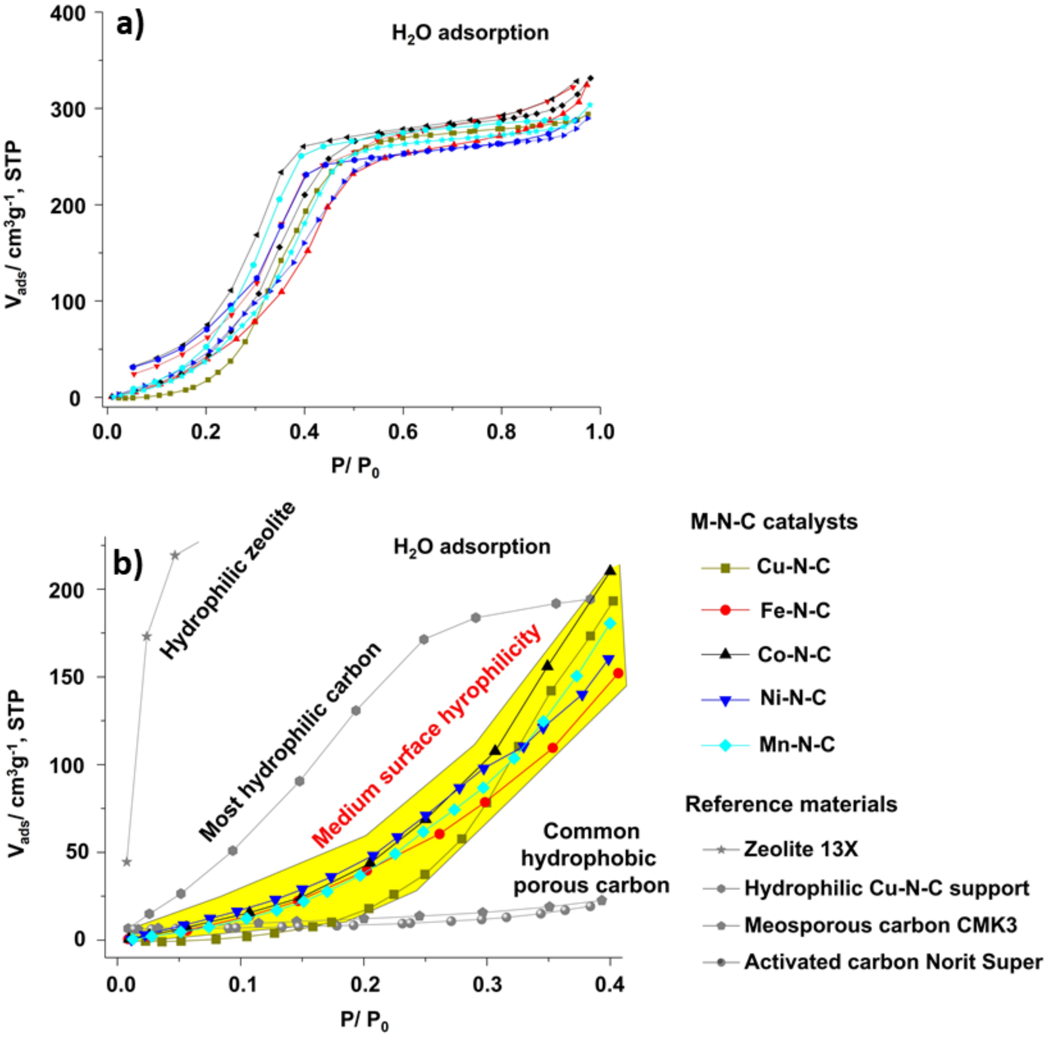

Supplementary Figure 4. a) Water vapor adsorption isotherms for this family of M-N-C electrocatalysts. b) Water vapor adsorption isotherms with highlighted low pressure regime together with typical benchmark samples, including hydrophilic zeolite 13X, most hydrophilic carbon as well as porous carbons (mesoporous CMK-3, commercial activated carbon Norit Super) that commonly show hydrophobic nature. As shown, the surface of this family of M-N-C is medium hydrophilic or hydrophobic as compared with the typical benchmark materials.

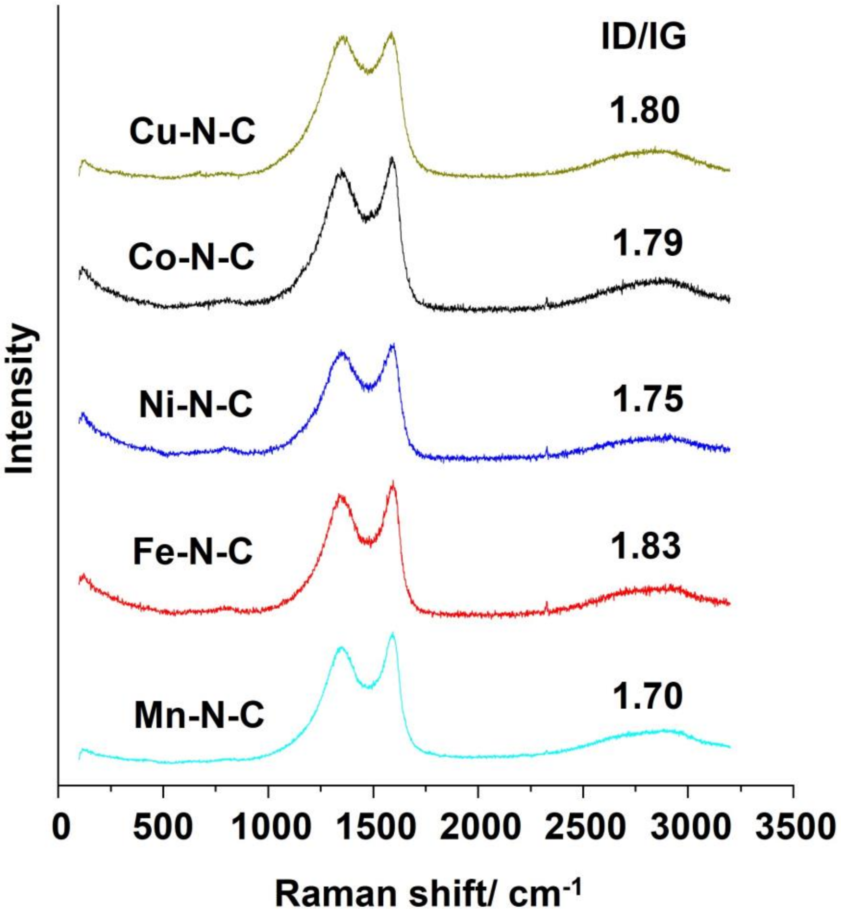

Supplementary Figure 5. Raman spectra of this family of M-N-C electrocatalysts. ID/IG ratio is based on peak fitting as shown in Table S2. As shown, the ratio is very close from one to another, indicating the density of defect sites of M-N-C is very similar.

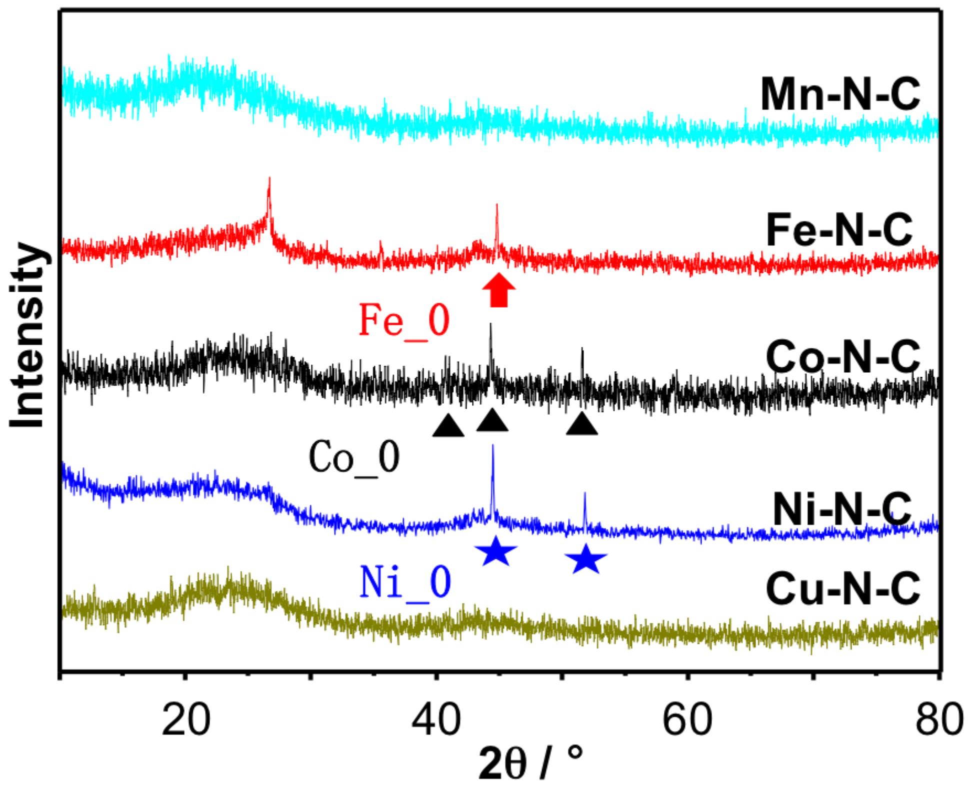

Supplementary Figure 6. XRD patterns of this family of M-N-C catalysts.

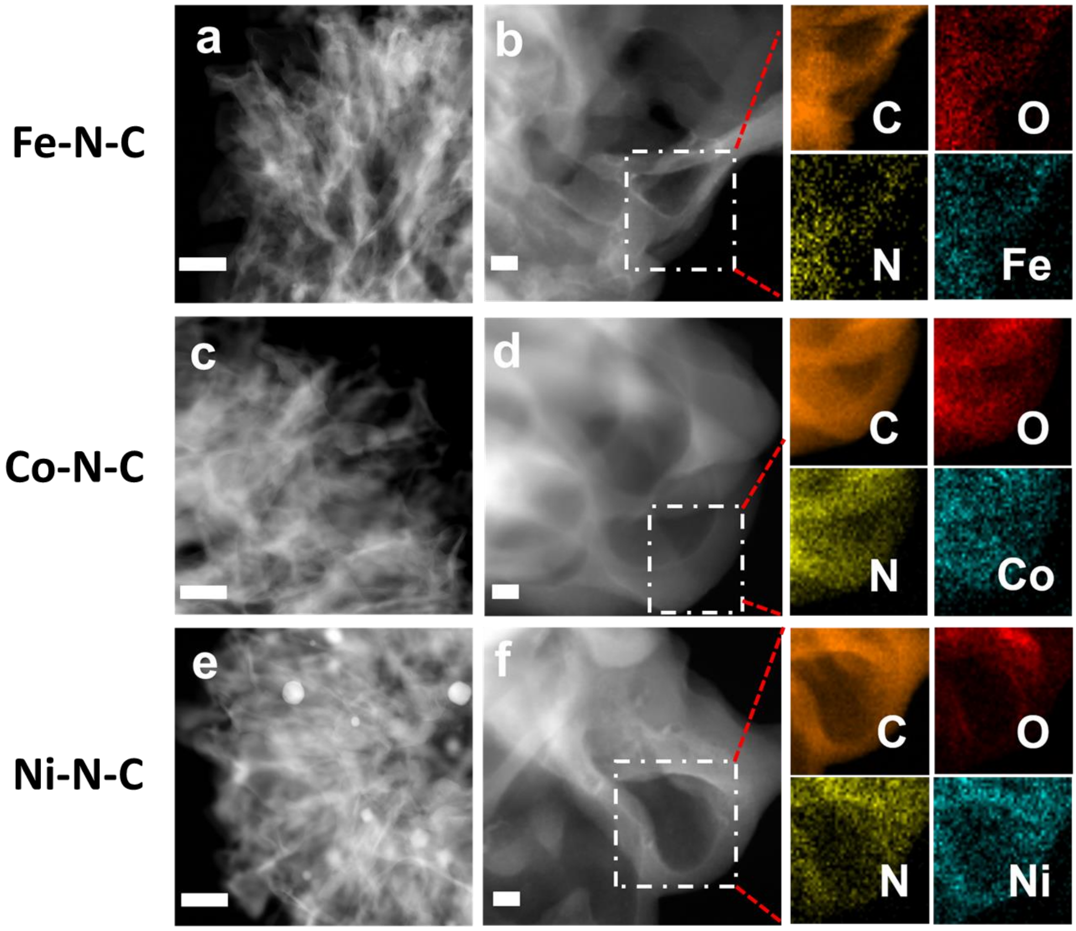

Supplementary Figure 7. STEM images and elemental maps for a,b) Fe-N-C, c,d) Co-N-C and e,f) Ni-N-C; Scale bar in left a,c,e) column: 200 nm, middle b,d,f)column: 20 nm.

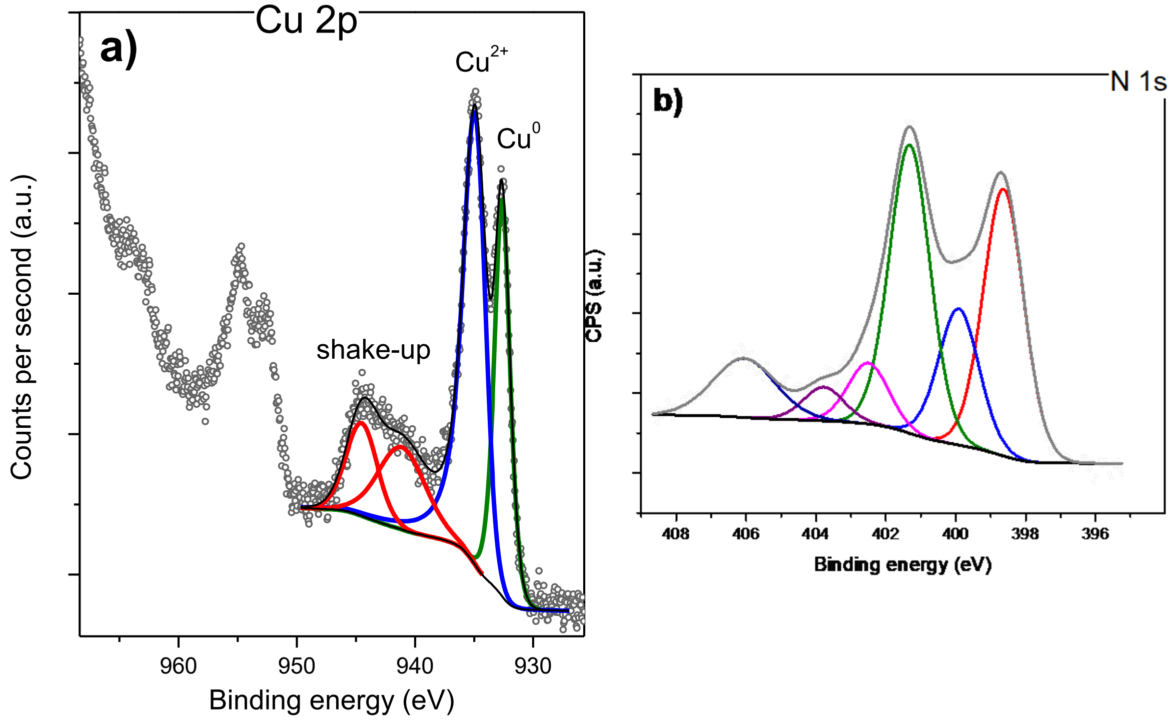

Supplementary Figure 8. High-resolution Cu 2p (a) and N 1s (b) XP spectra of the Cu-N-C sample. See main text for N 1s peak assignment.

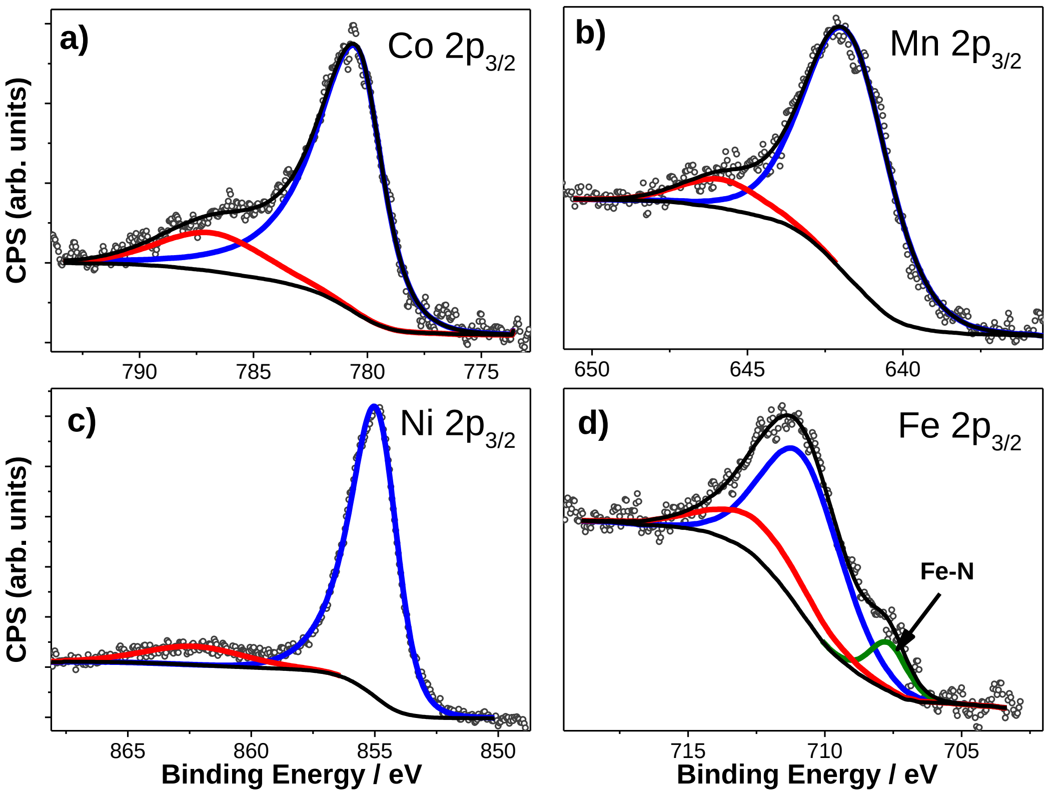

Supplementary Figure 9. High-resolution metal 2p3/2 of Co-N-C (a), Mn-N-C (b), Ni-N-C (c) and Fe-N-C (d). Blue lines represent 2p3/2 main peaks, red – the corresponding shake-up satellites.

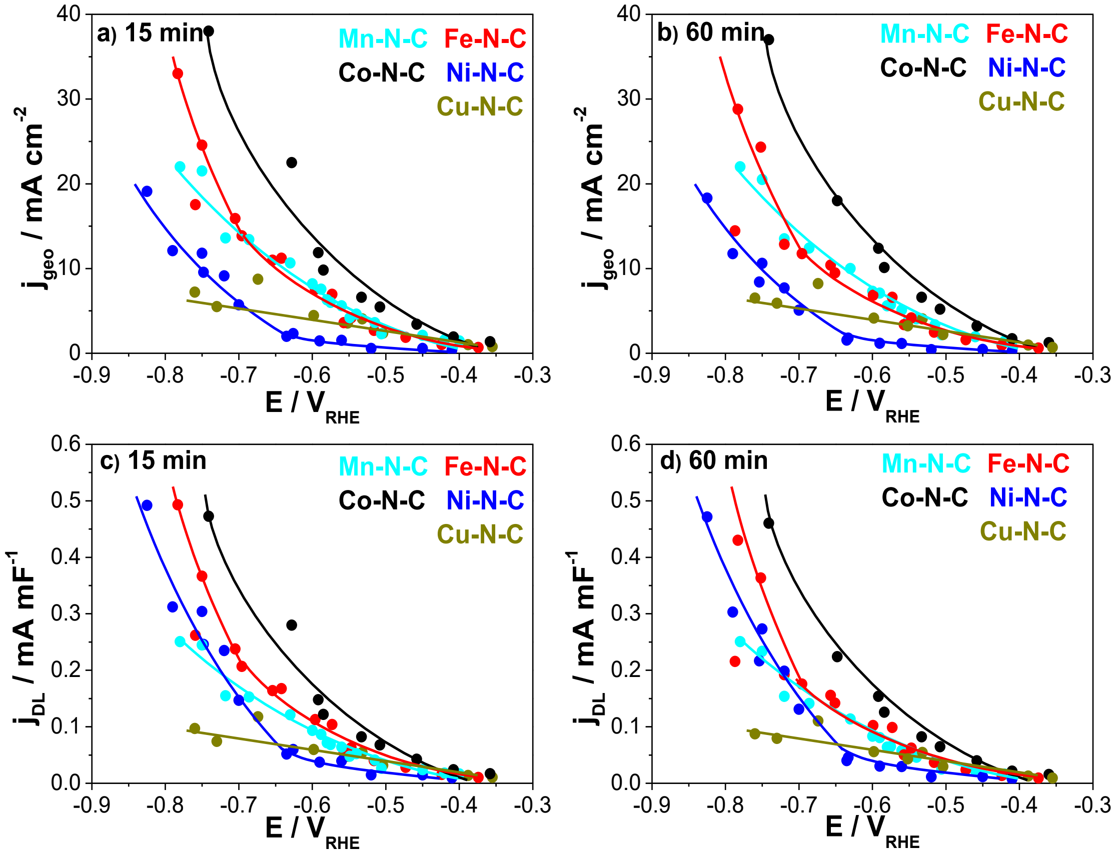

Supplementary Figure 10. Stationary potentiostatic catalytic activity of Cu-N-C (grey), Fe-N-C (red), Ni-N-C (blue), Mn-N-C (cyan) and Co-N-C (black) catalysts during bulk CO_2 electrolysis. Geometric current densities at a) 15 min b) 60min. Double layer capacity normalized stationary current densities: c) 15 min d) 60 min. Lines to guide the eye. Conditions: CO_2-saturated 0.1 M $KHCO_3$, 0.76 mg cm^{-2} catalyst loading

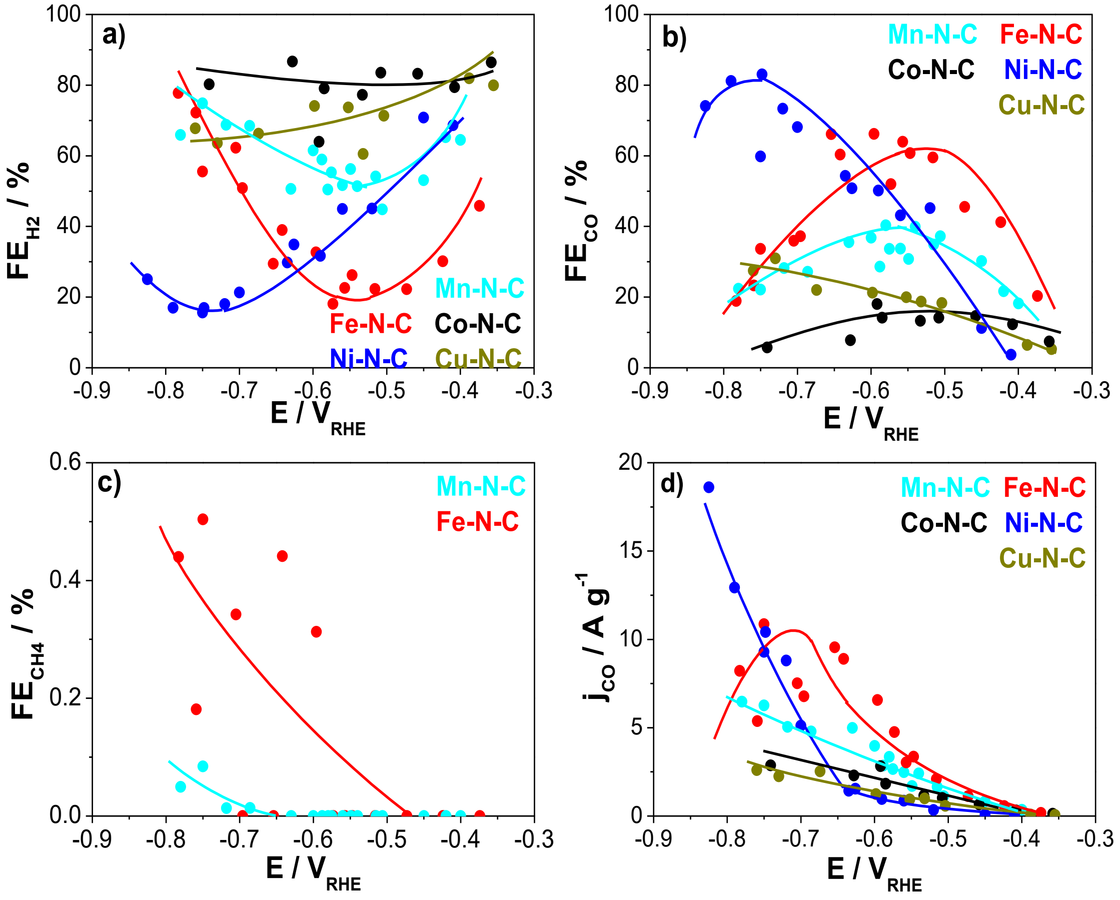

Supplementary Figure 11. Product efficiencies and yields. CO a) Faradaic efficiency and Absolute value of geometric reduction current density during bulk CO_2 electrolysis on Cu-N-C (grey), Fe-N-C (red), Ni-N-C (blue), Mn-N-C (cyan) and Co-N-C (black). Lines to guide the eye. Conditions: 15 min at constant electrode potential in CO_2-saturated 0.1 M $KHCO_3$ at 0.76 mg cm^{-2} catalyst loading.

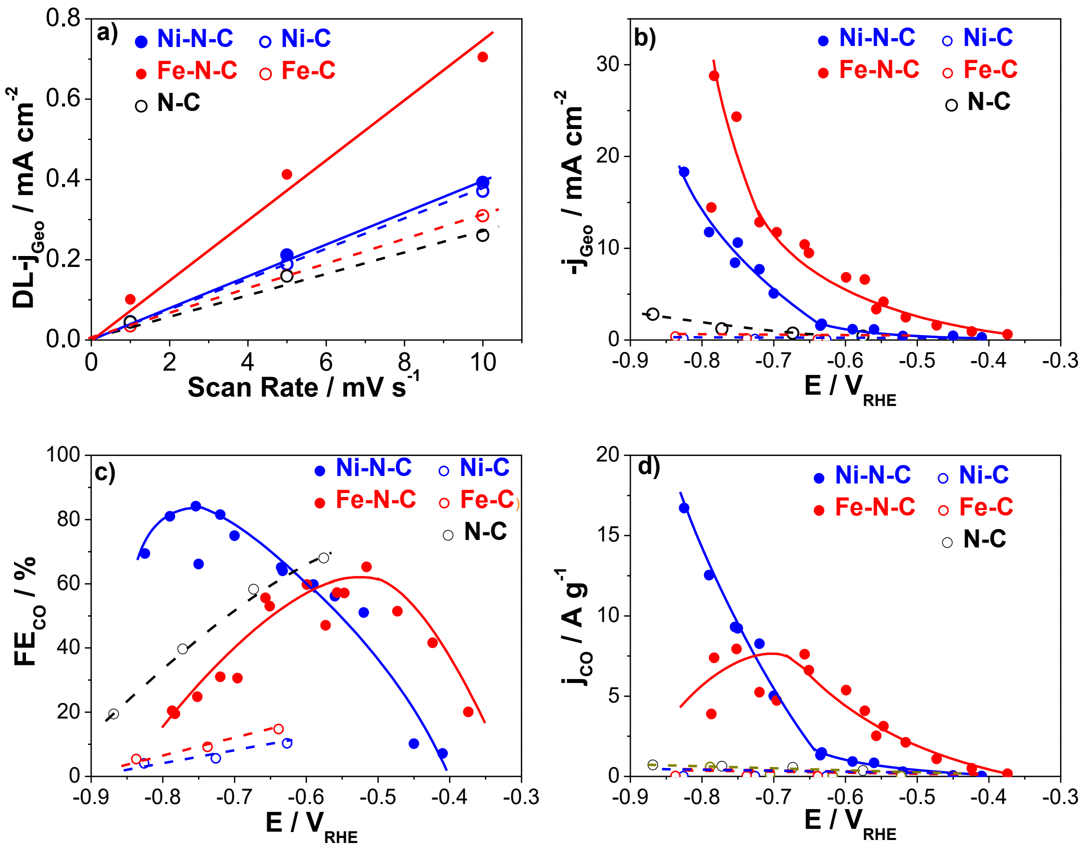

Supplementary Figure SI 12. Catalytic performance of Ni-N-C, Ni-C, Fe-N-C, Fe-C and N-C for CO2RR in 0.1 M CO_2 saturated $KHCO_3$ electrolyte, a) Double layer capacity, b) Absolute total geometric current density, c) Faradaic Efficiency towards CO and d) Mass normalized CO partial current density at 60 min of CO_2 bulk electrolysis. Guide for the eye lines are shown. Catalyst loading: 0.76 mg cm^{-2}.

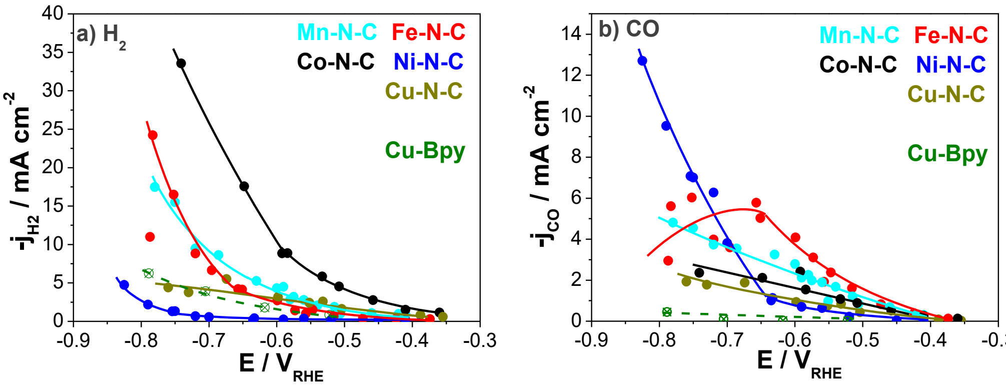

Supplementary Figure 13. Catalytic performance of Mn-N-C, Fe-N-C, Co-N-C, Ni-N-C, Cu-N-C and Cu-Bpy for CO2RR in 0.1 M CO_2 saturated $KHCO_3$ electrolyte. Geometric partial current density for a) H_2 and b) CO at 60 min of CO_2 bulk electrolysis. Lines to guide the eye. Catalyst loading: 0.76 mg cm^{-2}.

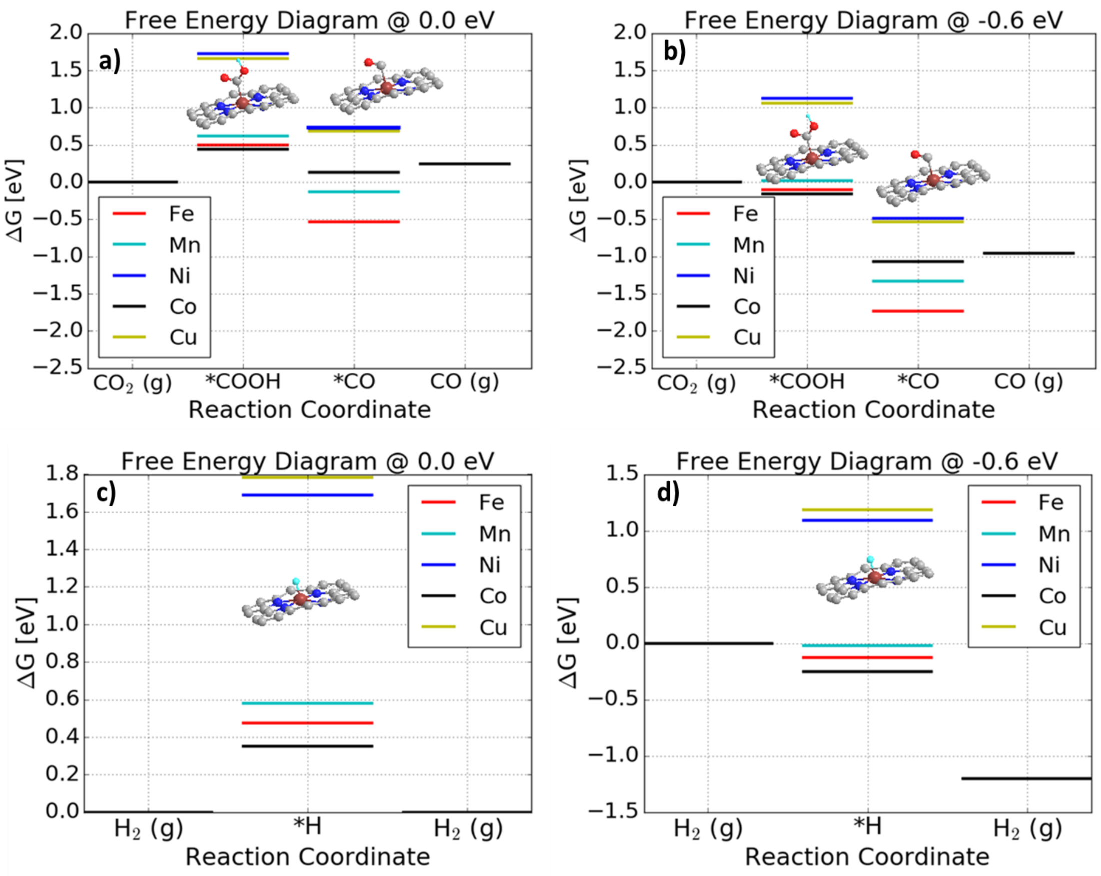

Supplementary Figure 14. DFT-calculated free energy diagram for the CO_2 reduction reaction (CO2RR) (a,b) and hydrogen evolution reaction (HER) (c,d) at 0 V_{RHE} (a,c) and -0.6 V_{RHE} (b,d).

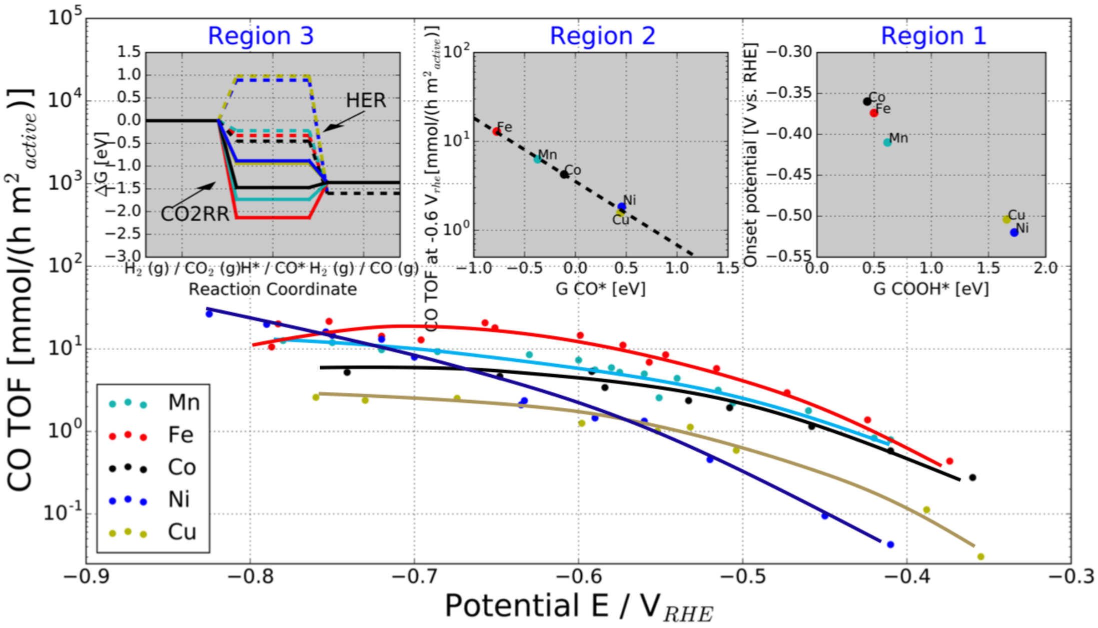

Supplementary Figure 15. Experimental CO production turnover frequency (TOF) of the M-N-C catalysts versus applied IR-corrected electrode potential (see Supplementary Equation 3). The catalytic reactivity trends split into 3 potential regions with distinctly different rate-determining mechanistic features. Insets: Region 1: Low overpotentials, the experimental onset potential of CO production correlates with the binding energy of the reaction intermediate COOH*. Region 2: Intermediate over-potentials, CO production TOF at -0.6 V_{RHE} vs. free energy of adsorbed CO, CO*; Region 3: High overpotentials, free energy diagrams for the HER (dashed paths) and CO2RR (solid paths) at -0.8 V_{RHE} for each M-N-C catalyst. HER barriers are high for Ni and Cu, while CO2RR is downhill making these materials favorable CO producing catalysts.

Supplementary Equations

Faradaic Efficiency

$$FE = \frac{\dot{V} * C * z * F}{R * j_{total}}$$ **Supplementary Equation 1**

FE: Faradaic Efficiency of the product / **%**

$\dot{V}$*: CO_2 gas flow rate /* **L s^{-1}**

C: Concentration of the product detected by GC / **Vol%**

z: Charge transfer of the product

F: Faradaic Constant / **C mol^{-1}**

R: Gas Constant / **22.4 L mol^{-1}**

j_{total}*: Total current during CO_2 bulk electrolysis /* **A**

CO-mass activity

$$j_{CO} = \frac{FE_{CO} * j_{geo}}{M}$$ **Supplementary Equation 2**

j_{CO}*: CO partial current density based on mass of catalyst /* **A g^{-1}**

FE_{CO}*: CO Faradaic Efficiency /* **%**

j_{geo}*: Geometric Current Density /* **A cm^{-2}**

M: Catalyst loading / **g cm^{-2}**

CO-geometric activity

$$Geo\ j_{CO} = \frac{\dot{V} * C * z * F}{R}$$ **Supplementary Equation 3**

$Geo\ j_{CO}$*: CO partial current density based on geometric area / m***A cm^{-2}**

$\dot{V}$*: CO_2 gas flow rate /* **L s^{-1}**

C: Concentration of the product detected by GC / **Vol%**

z: Charge transfer of the product

F: Faradaic Constant / **C mol^{-1}**

R: Gas Constant / **22.4 L mol^{-1}**

Evaluation of CO production turnover frequency

$$TOF = \frac{\dot{V} * C}{R * M * A_{BET} * x_{Metal-N}}$$ **Supplementary Equation 4**

TOF: BET Area and XPS Surface Metal-Nitrogen mole fraction Normalized CO generation rate

$\dot{V}$: *CO_2 gas flow rate /* ***L s^{-1}***

C: Concentration of the product detected by GC / ***Vol%***

R: Gas Constant / ***22.4 L mol^{-1}***

M: *Catalyst loading /* ***g m^{-2}***

A_{BET}: N_2 physiosorption BET surface area/ ***m^2 g^{-1}***

$x_{Metal-N}$: XPS surface Mole fraction of Metal-Nitrogen

Supplementary References

1 M.C. Biesinger, B. P. P., L.W.M. Lau, A.R. Gerson, R.St.C. Smart. Resolving surface chemical states in XPS analysis of first row transition metals, oxides and hydroxides: Cr, Mn, Fe, Co and Ni. *Appplied Surface Science* **257**, 887-898 (2010).

2 S. Muralidharan, R. G. H. Satellite in the x - ray photoelectron spectra of metalloporphyrins. *Journal of Chemical Physics* **71**, 2970-2974 (1979).

3 H.W. Nesbitt, D. B. Interpretation of XPS Mn(2p) spectra of Mn oxyhydroxides and constraints on the mechanism of MnO2 precipitation. *American Mineralogist* **83**, 305-315 (1998).

4 Bai, Y. *Photoelectron Spectroscopic Investigations of Porphyrins and Phthalocyanines on Ag(111) and Au(111): Adsorption and Reactivity (Doctoral Dissertation)*, (2010).
